# Manure Compost Is a Potential Source of Tetracycline-Resistant *Escherichia coli* and Tetracycline Resistance Genes in Japanese Farms

**DOI:** 10.3390/antibiotics9020076

**Published:** 2020-02-11

**Authors:** Nobuki Yoshizawa, Masaru Usui, Akira Fukuda, Tetsuo Asai, Hidetoshi Higuchi, Eiryu Okamoto, Kanako Seki, Hideshige Takada, Yutaka Tamura

**Affiliations:** 1 Laboratory of Food Microbiology and Food Safety, School of Veterinary Medicine, Rakuno Gakuen University, Ebetsu 069-8501, Japan; nyoshizawa.vet@gmail.com (N.Y.); s20803152@g.rakuno.ac.jp (A.F.); tamuray@rakuno.ac.jp (Y.T.); 2 Microbiology Section, Osaka Institute of Public Health, Osaka 543-0026, Japan; 3 Department of Applied Veterinary Science, The United Graduated School of Veterinary Science, Gifu University, Yanagido 501-1193, Japan; tasai@gifu-u.ac.jp; 4 Laboratory of Veterinary Hygiene, School of Veterinary Medicine, Rakuno Gakuen University, Ebetsu 069-8501, Japan; higuchi@rakuno.ac.jp; 5 Laboratory of Environmental Microbiology, College of Agriculture, Food and Environment Sciences, Rakuno Gakuen University, Ebetsu 069-8501, Japan; okamotoe@rakuno.ac.jp; 6 Laboratory of Organic Geochemistry, Faculty of Agriculture, Tokyo University of Agriculture and Technology, Fuchu 183-8509, Japan; a008gc@yahoo.co.jp (K.S.); shige@cc.tuat.ac.jp (H.T.)

**Keywords:** antimicrobial resistance, manure compost, residual antimicrobials, tetracyclines

## Abstract

Manure compost has been thought of as a potential important route of transmission of antimicrobial-resistant bacteria (ARB) and antimicrobial resistance genes (ARGs) from livestock to humans. To clarify the abundance of ARB and ARGs, ARB and ARGs were quantitatively determined in tetracycline-resistant *Escherichia coli* (harboring the *tetA* gene)-spiked feces in simulated composts. In the simulated composts, the concentration of spiked *E. coli* decreased below the detection limit at day 7. The *tetA* gene remained in manure compost for 20 days, although the levels of the gene decreased. Next, to clarify the field conditions of manure compost in Japan, the quantities of tetracycline-resistant bacteria, tetracycline resistance genes, and residual tetracyclines were determined using field-manure-matured composts in livestock farms. Tetracycline-resistant bacteria were detected in 54.5% of tested matured compost (6/11 farms). The copy number of the *tetA* gene and the concentrations of residual tetracyclines in field manure compost were significantly correlated. These results suggest that the use of antimicrobials in livestock constitutes a selective pressure, not only in livestock feces but also in manure compost. The appropriate use of antimicrobials in livestock and treatment of manure compost are important for avoiding the spread of ARB and ARGs.

## 1. Introduction

Antimicrobials are used to treat bacterial infections in both humans and animals. In livestock animals, antimicrobials are also used as growth promoters in several countries, including Japan. The extensive use of antimicrobials in the veterinary fields has led to great selective pressure for the development of antimicrobial-resistant bacteria (ARB) and the acquisition of transmissible antimicrobial resistance genes (ARGs) [1]. ARB and ARGs derived from livestock animals can spread to humans via food, contact, and the environment [2], thus compromising the effective treatment of bacterial infections in humans and constituting an important public health concern.

Manure compost could serve as an important route for the transmission of ARB and ARGs from livestock to humans, as livestock feces, which are manure components, contain high levels of ARB and ARGs [3]. Manure compost is applied for soil amendment and contributes to the spread of ARB and ARGs in the environment [4]. Moreover, such applications can lead to ARB and ARGs contamination of vegetables, and consequently to the transmission of ARB and ARGs from livestock to humans via the food chain.

Various composting methods are used in different countries to ensure the destruction of pathogens present in livestock feces and manure [5]. The United States Environmental Protection Agency (EPA) has suggested that the manure temperature should be maintained at 55 °C for 3 days [6]. In Europe, slurry and biogas plants utilizing mesophilic anaerobic digestion are usually used to treat livestock feces; slurry is easy and inexpensive to treat, and biogas plants are environmentally pollution-free and can provide electric power [7]. However, the methods used in European countries do not raise the manure temperature above 55 °C, and thus cannot destroy pathogens [6,8]. In Japan, manure compost is mainly treated by aerobic composting, which raises the manure temperature via aerobic fermentation [8].

By performing appropriate aerobic composting, the abundance of the ARB (including several pathogens) and ARGs are usually decreased [9,10]. In addition, several reports have shown that the concentration of residual antimicrobials in manure decreases with an increase in manure temperature by aerobic composting [10]. However, the effects of composting on reducing ARB, ARGs, and residual antimicrobials varies among several studies [10], because the composting process is a complex microbial dynamic that depend on materials and conditions. Therefore, more information is required on the effect of aerobic composting in several conditions to ARB, ARGs, and residual antimicrobials. However, information has not been reported to date for Japanese manure compost. 

In this study, to clarify the abundance of ARB and ARGs in manure compost derived from pig feces during the process of aerobic composting, the concentrations of ARB (tetracycline-resistant bacteria) and ARGs (tetracycline resistance genes) were determined in spiked feces and simulated composts. Tetracycline resistance was examined in this study, as it is most commonly distributed in livestock farms around the world, including Japan [11]. In Japan, the sales amounts of tetracyclines (311,177 kg/year) for veterinary uses were higher than those of the other classes of antibiotics in 2018 [12]. The *tetA* gene was selected as it is commonly associated with tetracycline resistance in Gram-negative bacteria and frequently isolated from livestock [13,14,15]. In addition, to clarify the field conditions of manure compost in Japan, the levels of tetracycline-resistant bacteria, tetracycline resistance gene (*tetA*), and residual tetracyclines were determined using field manure compost obtained from Japanese livestock farms. 

## 2. Results

### 2.1. Bacterial Survival and Maintenance of Resistance Genes in Spiked Pig Feces at Several Temperatures

**Figure 1:**
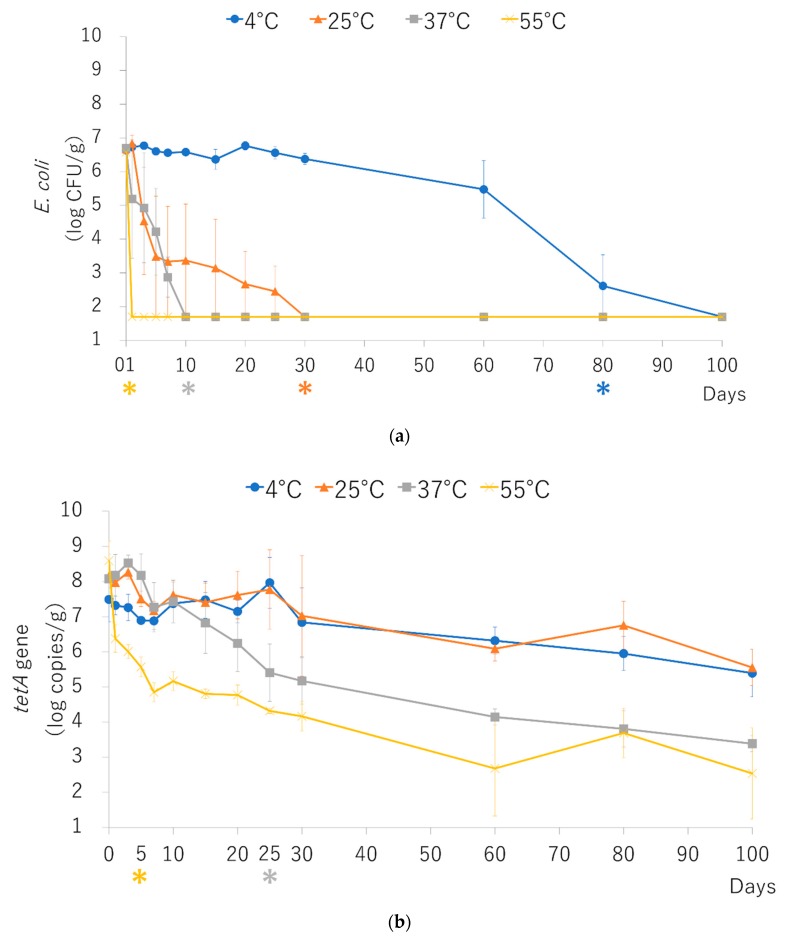
*E. coli* (**a**) and *tetA* gene (**b**) concentration during incubation at 4, 25, 37, and 55 °C in pig manure. Bars represent standard errors (*n* = 3). Asterisks (*) under the number of days represent the first day of significant difference compared to day 0 (*p* < 0.05). The detection limits are 50 CFU/g (*E. coli*) and 2.92 log copies/g (*tetA* gene).

*Escherichia coli* (TC7-1/DH5α) concentration decreased from the initial concentration to below the detection limit (50 CFU/g) at 55 °C one day after spiking, while at 37, 25, and 4 °C, the bacteria survived for 10, 30, and 80 days, respectively (Figure 1a). The concentration of *E. coli* significantly decreased after 1, 10, 30, and 80 days at 55, 37, 25, and 4 °C, respectively (*p* < 0.05). 

The *tetA* gene copy number significantly decreased from the initial copy number after 5 and 25 days at 55 and 37 °C, respectively (*p* < 0.05) (Figure 1b).

### 2.2. Bacterial Survival and Maintenance of Resistance Genes in Simulated Compost

**Figure 2:**
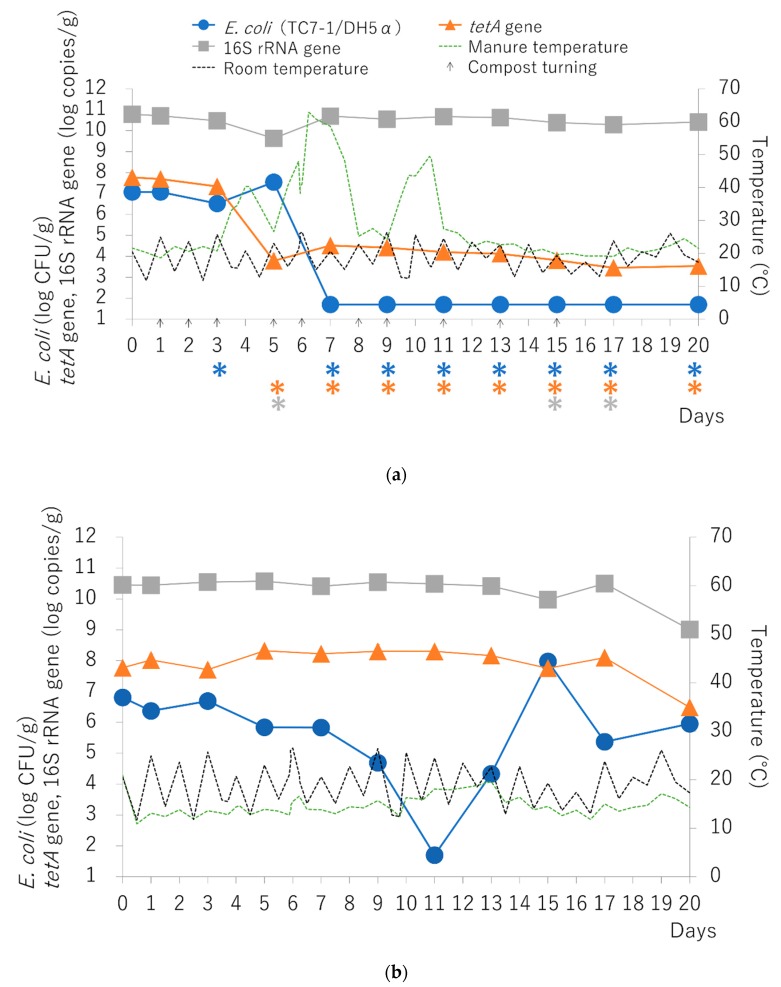
Variation in *E. coli*, *tetA* gene, and 16S rRNA gene amounts during simulated composting (**a**) and control (**b**) with pig manure. Bars represent standard errors. Asterisks (*) under the number of days represent the day of significant difference compared to day 0 (*p* < 0.05). The detection limits are 50 CFU/g (*E. coli*), 2.92 log copies/g (*tetA* gene), and 3.53 log copies/g (16S rRNA).

During 20 days of aerobic composting, the manure temperature increased from 21.6 °C (day 0) to 40.4 °C at day 4, 62.8 °C at day 6, and 49.5 °C at day 10 (Figure 2a). *E. coli* samples were not recovered after day 7 (detection limit is 50 CFU/g). The *tetA* gene copy number significantly decreased from 7.8 log copies/g at day 0 to 3.8 log copies/g at day 5 and to 3.5 log copies/g at day 20 (*p* < 0.05). Although the 16S rRNA gene copy number was significantly decreased at days 5, 15, and 17 (*p* < 0.05), the gene copy number was relatively maintained throughout the experiment (Figure 2a). In the control, the manure temperature did not vary and the *tetA* and 16S rRNA gene copy numbers were maintained throughout the experiment (Figure 2b). The concentration of *E. coli* was decreased to the detection limit (50 CFU/g) at day 11. After day 11 and until day 15, *E. coli* populations were increased and then decreased again.

### 2.3. Detection of Tetracycline-Resistant Bacteria and Tetracycline Resistance Genes from Field Manure Compost

Oxytetracycline-resistant bacteria (TCr) were detected in 54.5% (6/11) (pig farm, 57.1% (4/7), 3.4-4.4 log colony forming units (CFU)/g; cow farm, 50.0% (2/4), 4.2–4.6 log CFU/g; Table 1) of field manure composts. The *tetA* gene was detected in all farm samples (11/11; pig farm, 4.2-8.6 log copies/g; cow farm, 2.8–5.9 log copies/g).

### 2.4. Relationships between ARG Copy Number and Antimicrobial Concentration

**Figure 3:**
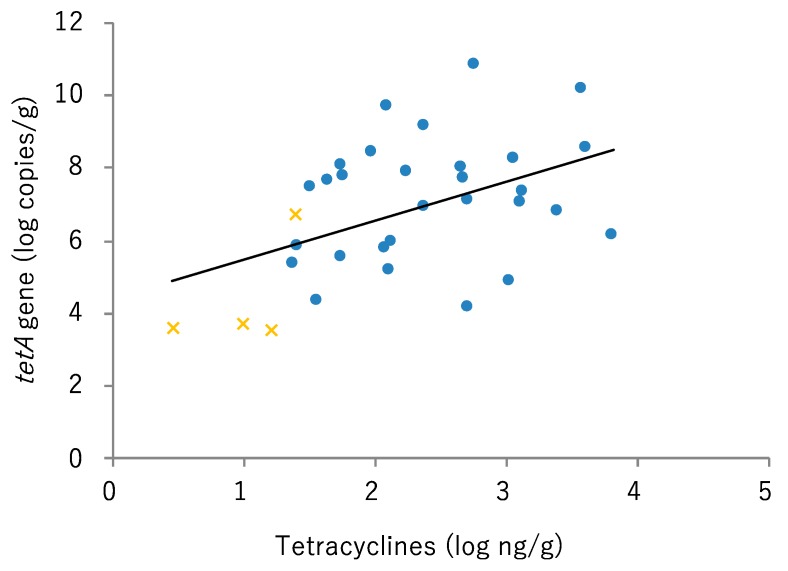
Relationship between *tetA* gene copy number and concentration of residual tetracyclines in manure compost. Manure compost derived from pig farm: blue dot; manure compost derived from cow farm: orange cross.

Tetracyclines (tetracycline, oxytetracycline, doxycycline, minocycline, and chlortetracycline) were detected in all tested manure compost samples (29 samples derived from the pig farm and 4 samples derived from the cow farm). The concentrations of residual tetracyclines in manure derived from pig samples were significantly higher than those from the cow samples (*p* = 0.00007331). Moreover, there was a significant correlation between the copy number of *tetA* (y = 1.0729x + 4.4226, R = 0.47, *p* = 0.005590) and the concentrations of tetracyclines (Figure 3).

## 3. Discussion

Most of the spiked *E. coli* decreased below the detection limit (50 CFU/g) following incubation at 55 °C within one day. This result suggests that most of the tetracycline-resistant *E. coli* decreased during manure composting using the temperature conditions recommended by the EPA (55 °C for three days) [6]. On the other hand, the spore-forming bacterium *Clostridium* (*Clostridioides*) *difficile* can survive in manure at 55 °C for 10 days [16]. Therefore, complete elimination of pathogens by aerobic composting is difficult; however, our results suggest that appropriate composting can decrease the concentration of tetracycline-resistant *E. coli*.

The spiked *E. coli* survived at 37, 25, and 4 °C for 7 to 100 days. The survival concentration of *E. coli* was higher at low temperatures. Some farms store the manure for composting for several days in Japan. Therefore, this holding time before composting could serve as a source of concentration of ARB in the environment, especially during the cold season. To avoid the spread of ARB into the environment, early and appropriate treatment of manure is required.

Under simulated composting conditions, spiked tetracycline-resistant *E. coli* was not detected after seven days, whereas TCr was detected in 54.5% of field manure compost samples. Temperature elevation during the composting is an important factor to reduce ARB in manure and aeration is necessary for that process. Inadequate conditions during composting, such as excess manure water content, excess or lack of aeration, and a cold climate, promote the long-term survival of ARB by preventing an increase in manure temperature during aerobic composting. If manure compost is not treated appropriately in livestock farms prior to being applied to agricultural land, it could serve as a transmission source of ARB and ARGs. In order to increase the temperature of the manure to reduce the concentration of ARB, it is necessary to turn the manure, extend the composting term, and then heat the manure.

High concentrations of ARGs are thought to be a risk to public health because ARGs from manure compost can transfer into human pathogens in soil [4,17]. The *tetA* gene remained in manure compost at 55 °C for 100 days of incubation and for 20 days during simulated composting, although the gene levels decreased. It was reported that the *tetA* gene remained in slurry after treatment at 55 °C for five days during anaerobic digestion of waste water solids and at 55 °C for 48 days during aerobic composting in an incubator [18,19]. Moreover, tetracycline resistance genes, including *tetA*, remained in manure after autoclave treatment (121 °C for 30 min, three times) [20]. In fact, TCr and *tetA* were detected in 54.5% and 100% of field manure compost, respectively (Table 1). These results suggest that ARB and ARGs are not eliminated in Japanese manure by composting, although they might decrease from the original stock. 

The concentrations of residual tetracyclines were higher in field manure compost derived from pigs than in that from cows. In addition, the ARG copy number and the concentrations of residual antibiotics in field manure compost were significantly correlated (Figure 3). It was reported that a higher amount of tetracycline is used in pig farms than in cattle farms and that tetracycline resistance rates of *E. coli* isolated from pig feces are higher than those from cattle feces in Japan [21]. Contrasting our results with these reported facts, it can be supposed that using antibiotics in livestock farms should cause a selective pressure for bacteria, not only in livestock feces but also in manure compost. Manure compost constitutes a transmission source of ARB and ARGs from livestock to the environment. The appropriate use of antibiotics in livestock and the treatment of manure compost are important processes to avoid the spread of ARB and ARGs. Moreover, more research on appropriate and effective conditions in composting processes is necessary, along with the development of composting systems to reduce the risk of ARB and ARG dissemination and to diminish residual antibiotics.

In conclusion, appropriate composting can reduce the concentrations of ARB and ARGs, although the elimination of ARB and ARGs is difficult. Indeed, most Japanese manure compost contains ARB and ARGs. This is the first report to specify the actual conditions in Japanese manure composts.

## 4. Materials and Methods

### 4.1. Bacterial Survival and the Maintenance of Resistance Genes in Pig Feces at Various Temperatures

The pig feces used in this study were collected from the pig farm at Rakuno Gakuen University (Hokkaido, Japan), stored at 4 °C, and used within three days after collection. Ethical authority was not required according to the Epidemiological and Animal Ethical Research Committee of Rakuno Gakuen University because fallen feces were collected. *E. coli* (TC7-1/DH5α) [22] harboring the *tetA* gene on a plasmid with chromosomal-encoded resistance to rifampicin (rifampicin minimum inhibitory concentration (MIC): 512 mg/L) was used as the bacterial inoculum. This bacterium is a common pathogen to humans from natural environments [17]. The strain was pre-cultured in Mueller–Hinton broth (OXOID, Hampshire, UK) at 37 °C for 24 h. Next, the pre-cultured strain was inoculated into 10 g of pig feces (final bacterial concentration: 10^6^~10^7^ CFU/g) in a tube. The plastic tube was kept at 4, 25, 37, and 55 °C in an incubator for 100 days. Samples were collected from the same tube on days 0, 1, 3, 5, 7, 10, 15, 20, 25, 30, 60, 80, and 100. Samples were mixed well before sampling. The test was conducted in triplicate.

To determine the concentration of bacteria in pig feces, 0.2 g samples were suspended in 1 mL of 0.85 % sterilized saline and serially diluted. Dilutions were plated on selective plates and incubated at 37 °C for 24 h. The selective plates consisted of Mueller–Hinton agar (OXOID, Hampshire, UK) supplemented with 50 mg/L of rifampicin (Sigma-Aldrich, St. Louis, MO, USA) for *E. coli* (TC7-1/DH5α). Incidentally, we confirmed that there was no growing colony on the selective plate by culturing unspiked pig feces used in this experiment.

The copy number of *tetA* gene in *E. coli* (TC7-1//DH5α)-spiked samples was determined by quantitative PCR (qPCR). The standard curves were generated by cloning the *tetA* gene (amplified from the *E. coli* (TC7-1/DH5α) using the primers detailed in Table S1) into the pTA2 vector and transforming them into *E. coli* DH5α competent cells (TaKaRa, Shiga, Japan) using the Target Clone kit (Toyobo, Osaka, Japan). Transformants harboring the recombinant plasmid were selected and the plasmids were extracted using the QIAprep Spin Miniprep Kit (Qiagen, Hilden, Germany) according to the manufacturer’s protocol. The concentration of the purified recombinant plasmid DNA in the samples was determined using a spectrophotometer (BioSpectrometer; Eppendorf, Hamburg, Germany) and the plasmids were then used as the qPCR standards. To determine the gene copy number in the samples, DNA was extracted from each 0.2 g of *E. coli* (TC7-1//DH5α)-spiked sample using the ISOFECAL kit (NIPPON GENE, Tokyo, Japan) according to the manufacturer’s protocol. Then, qPCR was performed using SYBR Premix Ex Taq II (Tli RNaseH Plus; TaKaRa, Shiga, Japan) in 20 μL reactions containing 5 μL of DNA template and 0.4 μM of each primer (Table S1). Analyses were performed using a Light Cycler 480II and software (Roche-Diagnostics, Basel, Switzerland). Reaction conditions included initial denaturation at 95 °C (30 s), followed by 45 cycles of 95 °C (3 s) and 52 °C for 30 s (Table S1). Melting curves were generated and analyzed to verify that no non-specific amplification had occurred. Analyses were performed three times in duplicate and the mean and standard errors were calculated.

### 4.2. Bacterial Survival and Maintenance of Resistance Genes in Simulated Compost

Pig feces (1877 g; moisture content: 66.64%), rice husk (623 g; moisture content: 9.58%), and 500 mL of an *E. coli* (TC7-1/DH5α) suspension (moisture content: 97.94%) (final moisture content: 60%, final bacterial concentration of *E. coli*: 10^7^ CFU/g) were added to an aerated (0.5 L/min) simulating compost reactor (Kaguyahime; Figure S1; Fujihira industry, Tokyo, Japan) for composting and to a non-aerated plastic vessel for the control. The room and manure temperatures at the center of the reactor and the vessel were measured with thermometers (TR-52i; T & D, Nagano, Japan). The reactor was operated until the manure temperature stopped increasing (for 20 days) following the manufacturer’s protocol. On days 1, 2, 3, 5, 6, 8, 9, 11, 13, and 15, the content of the reactor was turned to homogenize the compost and ensure aerobic conditions. Samples were collected on days 0, 1, 3, 5, 7, 9, 11, 13, 15, 17, and 20. Two reactors were operated simultaneously. The concentrations of inoculated *E. coli* and *tetA* gene were determined as described above. In addition, the concentration of 16S rRNA gene was determined by qPCR, similar to the above method used for the *tetA* gene with slight modifications (the annealing temperature was 52 to 60 °C; the primers are shown in Table S1). The construction of the standard curves of the 16S rRNA gene was done by the amplifying of *E. coli* gene (TC7-1/DH5α) with the primers indicated in Table S1.

### 4.3. Concentrations of Tetracycline-Resistant Bacteria and Tetracycline Resistance gene (tetA) in Field Manure Compost Samples

To analyze differences in the quantities of TC^r^ and ARGs among common farms in Japan, 11 field-matured manure compost samples (aerobic composting) were collected from 11 farms taken from cores of piles between August 2014 and November 2015 (seven from seven pig farms and four from four cow farms). The concentration of *tetA* gene was determined as described above. To determine the concentration of TCr, sample dilutions were spread on nutrient agar plates (Nissui Pharmaceutical, Tokyo, Japan) supplemented with 50 mg/L oxytetracycline (Sigma-Aldrich, City, MO, USA).

### 4.4. Relationship between the Concentration of Bacterial Genes and Residual Antimicrobials in Field-Matured Manure Compost Samples

To analyze the relationship between the ARG copy number and concentrations of antimicrobials in field manure compost, 33 field-matured manure compost samples (aerobic composting) were collected from 15 farms between August 2014 and November 2015 (29 samples from 11 pig farms, 4 samples from 4 cow farms). These samples were stored at −80 °C until use. The concentration of *tetA* gene was determined as described above. To measure the concentrations of tetracyclines, including tetracycline, oxytetracycline, doxycycline, minocycline, and chlortetracycline, the field manure compost samples were analyzed by liquid chromatography (Accela; Thermo Scientific, Waltham, MA, USA) with a tandem mass spectrometer (Liquid Chromatograph-tandem Mass Spectrometry (LC-MS/MS); Quantum Access, Thermo Scientific, Waltham, MA, USA) following extraction using a solid-phase cartridge (Oasis HLB resin; Waters Corp., Milford, MA, USA) [23].

### 4.5. Statistical Analysis

Dunnett’s test was used to determine dynamic changes in the relative abundances between inoculated bacteria and the *tetA* gene following incubation and composting compared to day 0. The Shapiro–Wilk test and Wilcoxson rank sum test were used to compare TCr and the *tetA* gene abundances between pig farms and cow farms. The Shapiro–Wilk test and Wilcoxon rank sum test were used to compare the concentrations of residual tetracyclines in field manure compost samples between pig farms and cow farms. These statistical analyses were performed using the R software. Pearson’s test was used to analyze correlations between the copy numbers of antimicrobial resistance genes and antimicrobial concentrations. Differences were considered significant at *p* < 0.05.

## Figures and Tables

**Table 1:** The prevalence and concentrations of TCr^a)^ and *tetA* in livestock manure

Manure Derived from	Farm ID	TCr (log CFU/g) ^b^	*tetA* (Log Copies/g) ^c^
Pig farm	A	4.35	4.35
	B	UD ^d^	5.82
	C	4.03	5.19
	D	3.38	8.56
	E	UD	6.00
	F	UD	4.18
	G	3.42	4.87
Cow farm	H	4.64	2.91
	I	4.18	2.75
	J	UD	5.90
	K	UD	2.79

^a^ TCr, oxytetracycline-resistant bacteria; ^b^ detection limit of TCr (log CFU/g) in manure is <50 CFU/g; ^c^ detection limit of *tetA* gene is < 2.92 log copies/g; ^d^ UD, below the detection limit.

## Supplementary Materials

The following are available online at https://www.mdpi.com/2079-6382/9/2/76/s1: Figure S1: Diagram of simulating compost reactor (Kaguyahime). Table S1: Primers used in this study.
