# Supplementary material for: Manure Compost Is a Potential Source of Tetracycline-Resistant Escherichia coli and Tetracycline Resistance Genes in Japanese Farms
Source: Antibiotics (Basel). 2020 Feb 11;9(2):76. doi: 10.3390/antibiotics9020076 (PMC7168215; doi:10.3390/antibiotics9020076)
Supplement: Supplementary file 1 [file antibiotics-09-00076-s001.zip › S1 Table(200208NY).docx]

**Table S1. Primers used in this study**

| Target | Purpose | Forward or Reverse | Sequence (5′-3′) | Tm (°C) | Product size (bp) | Reference |
| --- | --- | --- | --- | --- | --- | --- |
| *tetA* gene-speciﬁc primers | *tetA*  for standard | Forward | GCGCCTTTCCTTTGGGTTCTC | 60 | 1,033 | [1] |
|  |  | Reverse | CGTGATCGGGAGTATCTGGCTG |  |  |  |
| 63f-1387r | 16S rRNA  for standard | Forward | CAGGCCTAACACATGCAAGTC | 55 | 1,325 | [2] |
|  |  | Reverse | GGGCGGWGTGTACAAGGC |  |  |  |
| *tet(A)* | *tetA*  for qPCR | Forward | GCTACATCCTGCTTGCCTTC | 52 | 210 | [3] |
|  |  | Reverse | CATAGATCGCCGTGAAGAGG |  |  |  |
| 341F | 16S rRNA  for qPCR | Forward | CCTACGGGAGGCAGCAG | 60 | 192 | [4] |
| 534R |  | Reverse | ATTACCGCGGCTGCTGG |  |  |  |

**Supplementary References**

[1] Fukuda A, Usui M, Okamura M, Dong-Liang H, Tamura Y. The role of flies in the maintenance of antimicrobial resistance in farm environments. Microb Drug Resist 2019;25:127-32. https://doi.org/10.1089/mdr.2017.0371

[2] Marchesi JR, Sato T, Weightman AJ, Martin TA, Fry JC, Hiom SJ, Dymock D, Wade WG. Design and evaluation of useful bacterium-specific PCR primers that amplify genes coding for bacterial 16S rRNA. Appl Environ Microbiol 1998;64:795–9. Erratum in: Appl Environ Microbiol 64:2333.

[3] Ng LK, Martin I, Alfa M, Mulvey M. Multiplex PCR for the detection of tetracycline resistant genes. Mol Cell Probes 2001;15:209–15.

[4] Wei T, Miyanaga K, Tanji Y. Persistence of antibiotic-resistant and –sensitive *Proteus mirabilis* strains in the digestive tract of the housefly (*Musca domestica*) and green bottle flies (*Calliphoridae*). Appl Microbiol Biotechnol 2014;98:8357–66. https://doi.org/10.1007/s00253-014-5846-9
